# Immediate and early injection in unilateral vocal fold paralysis^☆^

**DOI:** 10.1016/j.bjorl.2019.11.001

**Published:** 2019-11-18

**Authors:** Geraldo Druck Sant'Anna

**Affiliations:** Universidade Federal de Ciências da Saúde de Porto Alegre (UFCSPA), Disciplina de Otorrinolaringologia, Porto Alegre, RS, Brazil

In most cases unilateral vocal fold paralysis occurs, secondary to traumatic, inflammatory, iatrogenic, neoplastic, neurological or idiopathic causes. It can result in significant negative effects on voice, swallowing and quality of life, and introduces a risk of aspiration pneumonia to patients affected by it.

The treatments currently available for this situation consist in promoting glottal coaptation. They are: speech therapy, vocal fold medialization, either by injection or thyroplasty (whether or not associated with arytenoid rotation) and reinnervation. These treatments may also be classified as temporary or definitive.^1^

When there is obvious injury to the motor innervation of the larynx, the accepted treatment is vocal fold medialization, with or without reinnervation, as a a definitive treatment. However, in patients who have the possibility of functional recovery, there is some controversy about what to do and when to do it.

Laryngeal injections, also called injection laryngoplasty, have become more popular in the last decade. This can be attributed to several factors: 1. new and safe injection materials, 2. the possibility of utilizing general or local anesthesia, and 3. the option of several approaches – cricothyroid membrane, thyroid notch, trans-thyroid and trans-oral cartilage. The evidence available through a meta-analysis does not show any significant differences between voice quality results when performed under general or local anesthesia.^2^

The materials that can be used and are available in Brazil are autologous fat, hyaluronic acid and hydroxyapatite. The fat needs to be removed from a donor area, and requires an incision. Hyaluronic acid is reabsorbed in approximately 60–180 days and hydroxyapatite, if injected into the superficial layer of the lamina propria, may impair mucosal vibration.

In recent years, publications have shown promising results with immediate or early injection (up to 6 months) of volatile substances in an attempt to avoid a definitive surgical treatment (thyroplasty). Four retrospective and prospective cohorts were grouped into a meta-analysis, which exhibited significant results. The analysis resulted in 275 grouped patients and the relative risk of not undergoing surgery was 0.25 (0.14-0.45), with a 95 % confidence interval. Therefore, injection laryngoplasty decreases the chance of requiring definitive surgery by up to 75 %. In other words, the chance of a patient undergoing definitive surgical treatment when the injection is not performed is up to 4-fold higher. There are limitations to this meta-analysis. The main one is that there is no randomized clinical trial to study the effect of injection laryngoplasty compared to voice observation and/or speech therapy because the studies are observational. Therefore, there could have been a selection bias favoring the hypothesis being assessed. Similarly, the voice analysis was not an outcome assessed in all studies and it cannot be stated whether or not patients who eventually were not submitted to thyroplasty accepted living with a worse voice. There is also no definition of the optimal timing for the injection – immediate (up to 3 months) or early (3–6 months). Nevertheless, the analyzed results were classified as degree of evidence C, allowing the recommendation of this treatment modality for paralysis, considering the current stage of scientific knowledge.

There is no explanation about the mechanism by which good vocal and swallowing quality would be maintained, even after resorption of the injected material. My hypothesis is that resorption occurs gradually, day after day, leading to a slow, gradual and consistent adaptation of the entire vocal tract to a new glottic configuration.

In Brazil, the strategy of immediate or early injection in unilateral vocal fold paralysis in patients with the possibility of functional recovery is not yet popular. This, in my understanding, leads to an unnecessary and difficult wait for a definitive treatment. There is no justification for a ‘wait and see’ strategy.^2^, ^3^ Patients face an increased risk of pulmonary complications, as well as to daily vocal and swallowing consequences caused by glottic insufficiency.

## Conflicts of interest

The author declares no conflicts of interest.
